# Clinical Pharmacokinetics of Enalapril and Enalaprilat in Pediatric Patients—A Systematic Review

**DOI:** 10.3389/fped.2021.611322

**Published:** 2021-02-12

**Authors:** Muhammad Faisal, Willi Cawello, Stephanie Laeer, The LENA Consortium

**Affiliations:** Institute of Clinical Pharmacy and Pharmacotherapy, Heinrich-Heine-University Düsseldorf, Universitätsstrasse 1, Düsseldorf, Germany

**Keywords:** enalapril, systematic review, pharmacokinetics, pediatrics, heart failure, hypertension, enalaprilat

## Abstract

**Purpose:** Enalapril has an established safety and efficacy in adults and is used in hypertension, heart failure, and renal failure. In pediatric patients, enalapril is labeled for children with hypertension and used off label in children with heart failure. The systematic literature search aims to assess the current knowledge about enalapril and its active metabolite enalaprilat pharmacokinetics in children as a basis for dose delineation for pediatric patients with heart failure.

**Methods:** A systematic literature review was performed in the PubMed database using relevant keywords. Dose normalization of relevant pharmacokinetic parameters of the identified studies was done for comparison between different diseases and pediatric age groups.

**Results:** The literature search has resulted in three pediatric pharmacokinetic studies of enalapril out of which Wells et al. reported about children with hypertension and Nakamura et al., and Llyod et al. presented data for pediatric heart failure patients. The area under the curve values of enalaprilat in hypertensive pediatric patients increased with respect to the age groups and showed maturation of body functions with increasing age. Dose normalized comparison with the heart failure studies revealed that although the pediatric heart failure patients of > 20 days of age showed the area under the curve a similar to that of hypertensive patients, two pediatric patients of very early age (<20 days) were presented with 5–6-fold higher area under the curve values.

**Conclusion:** Data related to the pharmacokinetics of enalapril and enalaprilat in hypertensive patients and few data for young heart failure children are available. Comparison of dose normalized exposition of the active metabolite enalaprilat indicated similarities between heart failure and hypertensive patients and a potentially high exposition of premature patients but substantially more pharmacokinetic studies are required to have reliable and robust enalapril as well as enalaprilat exposures especially in pediatric patients with heart failure as a basis for any dose delineation.

## Introduction

A pediatric population is a heterogeneous group with developmental differences in physiology, pathology, and biochemistry from birth until adolescence (1). Due to these differences, the pediatric age groups are arbitrarily sub-classified into preterm newborn, newborn (0–28 days), infant (>28 days−12 months), toddler (>12 months−23 months), preschool child (2–5 years), school-age child (6–11 years), and adolescents (12–18 years) (2, 3). Each sub-group should be considered as a special population and thorough testing of drugs encompassing all representative pediatric age groups shall allow adequate decision-making in pediatric dosing and avoid sub-therapeutic and adverse effects of drugs (3, 4).

If the disease reveals a similar etiology in adults and children meaning that the efficacy can be extrapolated from adulthood to childhood and if the safety information is sufficiently described, only pharmacokinetic investigations in all pediatric age groups remain to be performed to delineate adequate dosing information to capture the maturation processes of drug absorption, metabolism, and elimination (5, 6).

Enalapril is an angiotensin-converting enzyme inhibitor drug (7) used in adult patients with hypertension (8), heart failure (9, 10), and renal failure (11) and has established safety and efficacy in adults. In pediatric patients, however, it has only been labeled for infants and children with hypertension from 1 month of age (12). Enalapril is also prioritized to be used as the first-line treatment in pediatric heart failure and is together with captopril the most widely used off label drug in 70–80% of pediatric heart failure patients for more than 30 years (13, 14). Angiotensin-converting enzyme inhibitors (ACE) are recommended in international guidelines for pediatric heart failure due to several published non-randomized controlled clinical trials suggesting effective and safe use in children of all age groups (15) and high level of evidence of improved heart failure prognosis in adult heart failure use (13).

The safe and effective use of enalapril in pediatric patients needs a detailed evaluation of the pharmacokinetics of enalapril and its active metabolite enalaprilat in heterogeneous pediatric age groups (16). The systemic exposures of enalapril and enalaprilat may be influenced by the age-dependent maturating metabolizing carboxylesterases enzyme expressions in the liver (17) responsible for the conversion of enalapril to enalaprilat (18). Importantly, the kidney functions especially the rapidly maturating glomerular filtration rate (19) may influence the renal elimination of enalapril and enalaprilat especially in neonates (20). Also, the maturating expression levels of binding proteins (21) may influence the distribution of drug and metabolite in the tissues (1).

The disease state may alter the systemic exposure of enalapril and enalaprilat and shall require proper dose adjustments in pediatric patients. Pathophysiological effects of adult heart failure like systemic arterial hypoperfusion, neuro-hormonal activation, and venous congestion have reportedly altered the (22) absorption (23), distribution (24), renal and metabolic elimination (19) of drugs. The volume overload condition may cause pleural effusion, ascites, and anasarca, in adult patients and thus significantly alters the distribution of water-soluble drugs in these modified compartments (22). The clearance of enalapril and its active metabolite enalaprilat was reportedly lower in adult heart failure patients as compared to the adult hypertensive patients (25). Clinical presentations in pediatric heart failure like edema, growth failure, and circulatory derangements (26) may also alter the pharmacokinetics of drugs and may require dosing adjustments (27, 28). These pharmacokinetic changes may be indicated by the pharmacokinetic parameters like AUC, Cmax, Tmax, clearance, as well as half-life that can be useful for determining pathophysiological effects on the drug disposition and in adjusting the dose of drugs for safe and effective drug administration.

The present work was aimed at performing a systematic literature search to evaluate whether conclusive pediatric pharmacokinetic information of enalapril and its active metabolite enalaprilat in different pediatric age groups are available for understanding the effect of age on enalapril pharmacokinetics for children suffering from hypertension and heart failure.

## Methods

### Searching Criteria

**Figure 1:**
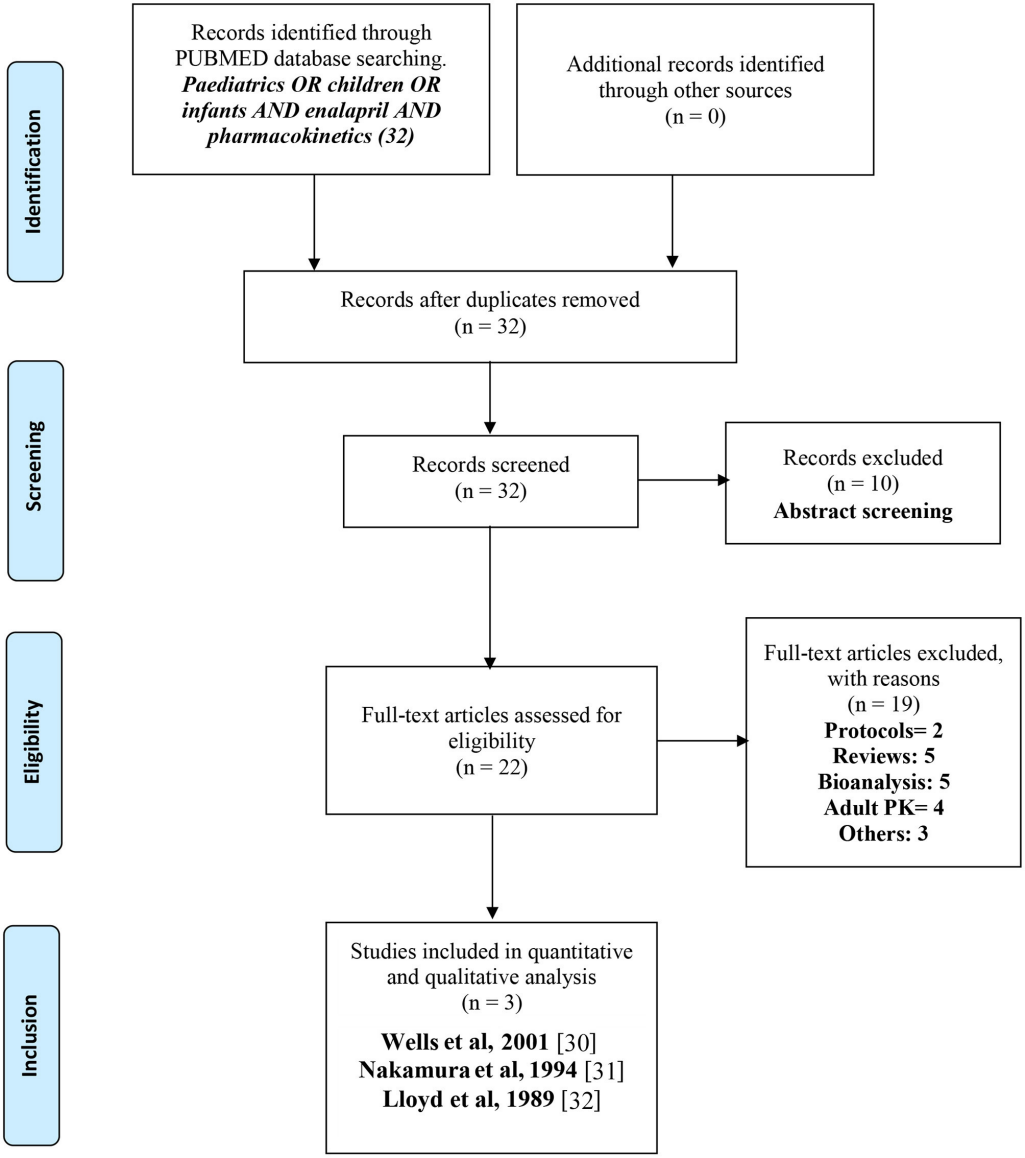
PRISMA (29) flow diagram for the systematic selection of pharmacokinetic data of enalapril in pediatric heart failure and hypertensive patients reported in the literature.

The PRISMA (Preferred Reporting Items for Systematic Reviews) guidelines were followed during the process of a systematic review as illustrated in Figure 1 (29). Studies with reported pharmacokinetic parameters of enalapril or enalaprilat in pediatric patients were screened and selected. Studies that were related to enalapril in healthy adults or adult patients with heart failure or hypertension were not included. Besides, the design/protocols of clinical trials were also excluded. Studies relating to other angiotensin-converting enzyme inhibitors or antihypertensive/cardioprotective drugs were excluded from the search. Initially, titles, abstracts, figures, and tables were screened to select or remove the studies that did not match the inclusion or exclusion criteria, respectively. In the next step, all the manuscripts were investigated thoroughly and were selected as per the inclusion criteria.

### Database Search

The systematic review was conducted using the PubMed database developed by the National Center for Biotechnology Information (https://www.ncbi.nlm.nih.gov/pubmed/) at the national library of medicine. No filters were applied on the searched or publication date. The reference lists of the selected searches were screened for the inclusion of additional articles according to the predefined eligibility criteria. The database search identified research items that were informative in terms of the pharmacokinetic parameters, enalapril concentrations in the treatment of pediatric heart failure and hypertension. The key words included for the search were (Pediatrics OR children OR infants) AND enalapril AND pharmacokinetics.

### Study Selection

The title of the searched items was screened for any duplication of items and potential relevance by the inclusion criteria. In the next step, the abstracts of the selected articles were screened. The abstracts were screened for information relating to the pharmacokinetic evaluations of enalapril in pediatric patients with heart failure or hypertension, and potential full papers were short-listed for further evaluations. In the last step, the articles were assessed for eligibility criteria and the articles with no pharmacokinetic data or enalapril concentrations in pediatric patients were excluded. The shortlisted full papers were further investigated and assessed for inclusion and retrieval of the required information.

### Data Collection Process

The full texts were investigated for the study design and the reported pharmacokinetic results were recorded in detail for each study. The data were further discussed and filtered for relevance and the collected data were included/tabulated in Table 1 for enalapril and Table 2 for enalaprilat and verified by all authors.

**Table 1:** Extracted enalapril pharmacokinetic parameters of pediatric patients reported in the literature.

**Source/study**	**Design**	**Age**	**Disease**	**Enalapril dose (mg/kg)**	***C*_**max**_ (ng/mL)**	***T*_**max**_ (h)**	Enalapril AUC_**0−24**_ (ng/mL^*^h)	***t*_**1/2**_ (h)**
Wells et al. (32)	SD MD	All 4 groups 1 month to 16 years (*n* = 40)	Hypertension	0.15 mg/kg^#1^ 2.5 or 5 mg^#2^	28.2–45.9 24.6–45.4	1	NR	NR
Nakamura et al. (1)	SD	<20 days (*n* = 3)	Heart failure	0.05–0.30 mg/kg^#3^	NR	8-12	268.7 (160.9–425.4)^#6^	10.3 (4.2–13.4)
	SD	>20 days up to 6.5 years (*n* = 11)	Heart failure	0.05–0.30 mg/kg^#3^	NR	2	82.7 (23.7–167.9)^#6^	2.7 (1.3–6.3)
	SD	21 to 39 years (*n* = 7)	Healthy volunteer	0.12–0.17 mg/kg^#5^	NR	NR	83.4 ± 8.6^#6^	1.4 ± 1.0
Lloyd et al. (2)	SD, 2nd dose and 3rd dose	6 weeks to 8 months (*n* = 10)	Heart failure	Days 1, 2, 3 0.02/0.04/0.08 mg/kg^#4^	NR	NR	NR	NR

*SD, single dose; MD, multiple dose; Cmax, maximum concentration; Tmax, time to get maximum concentration; AUC, area under the curve; t_1/2_, half-life*.

#1 *Liquid suspension 0.15 mg/kg for 1 month to 6 years age*.

#2 *2.5 mg tablet daily for <28 kg, 5 mg daily for weight >28 kg for 6 years to 16 years age*.

#3 *Oral suspension, extemporaneously prepared from commercial tablets*.

#4 *Lyophilized form of enalapril maleate powder administered as an oral solution*.

#5 *Tablet formulation*.

#6 *Reported for normalized dose of 0.1 mg/kg*.

**Table 2:** Extracted enalaprilat pharmacokinetic parameters of pediatric patients reported in the literature (30–32).

**Source/study**	**Design**	**Age**	**Disease**	**Enalapril dose**	**Enalaprilat *C*_**max**_ (ng/mL)**	***T*_**max**_ (h)**	Enalaprilat AUC_**0−24**_ (ng/mL^*^h)
Wells et al. (32)	SD MD	Group I (1–24 months, *n* = 9)	Hypertension	0.15 mg/kg^#3^	16.8 (11.2–25.2)^#5^ 19.5 (13.8–27.6)^#5^	5.98 (3.98–8.00)^#5^ 4.17 (4.05–6.01)^#5^	197.1 (137.8–281.9)^#5^ 235.6 (169.0–328.4)^#5^
	SD MD	Group II (25 months to 6 years, *n* = 9)	Hypertension	0.15 mg/kg^#3^	16.4 (10.9–24.7)^#5^ 27.6 (19.5–39.1)^#5^	5.04 (3.13–6.92)^#5^ 3.99 (3.02–4.05)^#5^	211.1 (147.6–302.0)^#5^ 317.0 (227.4–441.8)^#5^
	SD MD	Group III (6–12 years, *n* = 10)	Hypertension	2.5 or 5 mg^#4^	24.4 (16.6–35.8)^#5^ 34.1 (24.5–47.5) ^#5^	5.01 (4.01–6.03)^#5^ 3.05 (2.04–4.05)^#5^	264.4 (188.3–371.3)^#5^ 352.0 (256.9–482.4)^#5^
	SD MD	Group IV (12–16 years, *n* = 12)	Hypertension	2.5 or 5 mg^#4^	40.9 (28.3–59.1)^#5^ 47.7 (35.3–64.5)^#5^	4.00 (3.00–5.01)^#5^ 3.00 (2.00–4.00)^#5^	409.1 (295.9–565.5)^#5^ 464.8 (348.6–619.8)^#5^
Nakamura et al. (1)	SD	<20 days (*n* = 3)	Heart failure	0.05–0.30 mg/kg,	NR	4.00	691.5 (447.4–892.2)^#1^^,^ ^#7^
	SD	>20 days up to 6.5 years (*n* = 11)	Heart failure	0.05–0.30 mg/kg	9.0 ± 4.7^#1^^,^ ^#6^	7.3 ± 2.4^#6^	138.4 (43.4–285.3)^#1^^,^ ^#7^
	SD	21–39 years (*n* = 7)	Healthy adults	0.12–0.17 mg/kg	30.1 ± 14.0^#1^	3.7 ± 1.4^#6^	245.7 ± 61.8^#1^^,^ ^#6^
Lloyd et al. (2)	SD	6 weeks to 8 months (*n* = 10)	Heart failure	Day 1 (0.02 mg/kg)	2.1 ± 1.7^#6^	(4)^#2^	NR
	2nd dose	6 weeks to 8 months (*n* = 10)	Heart failure	Day 2 (0.04 mg/kg)	5.5 ± 1.6^#6^	(4)^#2^	NR
	3rd dose	6 weeks to 8 months (*n* = 10)	Heart failure	Day 3 (0.08 mg/kg)	12.7 ± 2.3^#6^	(4)^#2^	NR

*SD, single dose; MD, multiple-dose; Cmax, maximum concentration; Tmax, time to get maximum concentration; AUC, area under the curve; t_1/2_, half-life*.

#1 *Reported for normalized dose of 0.1 mg/kg*.

#2 *The time of mean concentrations (over time) but not mean of t_max_*.

#3 *Liquid suspension 0.15 mg/kg for 1 month to 6 years age*.

#4 *2.5 mg tablet daily for <28 kg, 5 mg daily for weight >28 kg for 6 years to 16 years age*.

#5 *Geometric (95% confidence interval)*.

#6 *Arithmetic mean ± SD*.

#7 *Arithmetic mean (range); n, number of profiles, e.g., Nakamura n = 3 means 3 profile: 2 times patient 1 and 1 profile patient 2 = 2 patients with age <20 days*.

### Data Extraction

The information related to the year of publication, study design (single or multiple dosing), age groups, number of patients, the disease state of patients, dose administered, were summarized in Tables 1, 2 for enalapril and enalaprilat respectively. Pharmacokinetic parameters of enalapril and enalaprilat including the area under the curve (AUC), maximum concentration achieved in plasma (Cmax), time to achieve maximum concentration (Tmax), elimination half-life (*t*_1/2_) were extracted.

### Normalization of Pharmacokinetic Parameters and Handling of Missing Data

**Figure 2:**
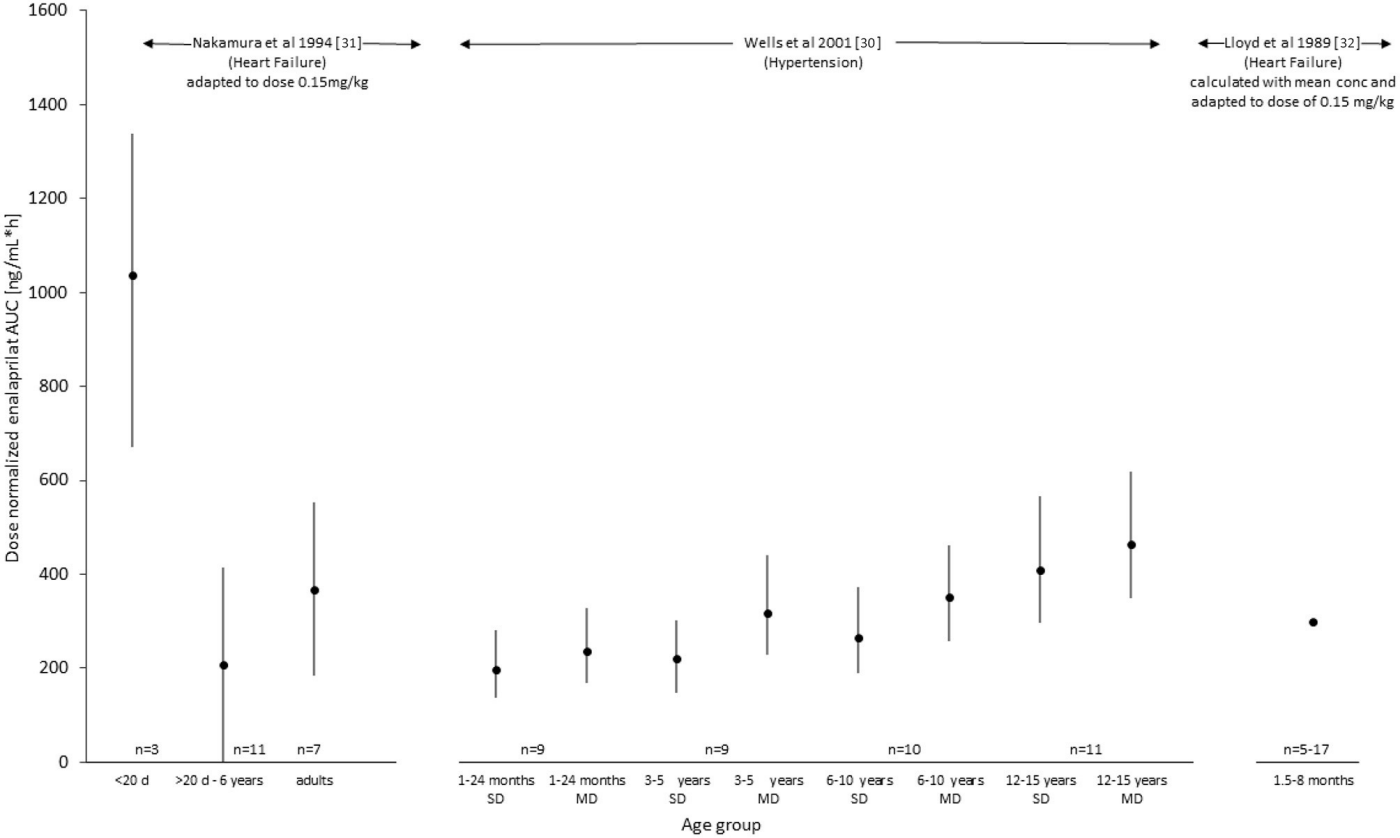
Exposure (AUC, ng/mL*h) of enalaprilat after administration of enalapril with a dose of 0.15 mg/kg. Values were taken out of the literature for Wells et al. (30) and dose-normalized for Nakamura et al. (31) as well as calculated out of the given plasma concentrations for Lloyd et al. (32) (see also Table 2).

To perform a comparison of the systemic exposure of enalaprilat between studies, the pharmacokinetic parameter of AUC was normalized to a comparable fictive dose of 0.15 mg/kg and reported in Figure 2. Whereas Wells et al. (30) reported statistics of AUC using the geometric mean combined with a 95% confidence interval, Nakamura et al. (31) reported arithmetic means with standard deviation and range. For the reason of comparability, the illustration (Figure 2) depicts dose-normalized AUC of Enalaprilat for Nakamura et al. (32) and arithmetic mean ± 2 times SD (corresponds to 95% of normal distributed values) and geometric means with 95% confidence intervals for Wells et al. (30) data. Because of only three plasma concentration profiles of two patients <20 days of age, these results are presented by the arithmetic mean and range. The paper of Lloyd et al. (32) has not presented results of enalaprilat AUC but the mean enalaprilat concentrations at time point 4, 6, 8 and 24 h. The concentrations obtained after the administration of 0.08 mg/kg of dose were normalized to the dose of 0.15 mg/kg and AUC was calculated with the mean of transformed concentrations. The dose-normalized AUC values of each study were illustrated in Figure 2 for a comparison of metabolite exposure in different age groups and disease states.

### Data Analysis

After the summarization of the data, pharmacokinetic information from the literature search was then evaluated to understand what level of information regarding absorption, disposition, and exposure of enalapril and its active metabolite enalapril was available in the heterogeneous pediatric age groups.

## Results

PubMed database search identified 32 articles, out of which 10 articles were excluded during the initial screening and only 22 articles were further evaluated. Based on the eligibility criteria, 19 articles were excluded and 3 articles were finally selected after deeper evaluations and the analysis of these articles was performed in this current study. Information like study authors, study design, age groups, disease state, dose and dosage form administered, and pharmacokinetic parameters of each pediatric age group was noted in tabular form and these parameters were then further discussed and compared to conclude the evidence related to exposure of enalapril and enalaprilat in pediatric patients. The screening process resulted in only three studies, i.e., Wells et al. (30), Nakamura et al. (31), and Llyod et al. (32) with reported pharmacokinetic parameters and serum plasma concentrations. Nakamura et al. (31) and Llyod et al. (32) reported the pharmacokinetics of enalapril in pediatric heart failure and Wells et al. (30) reported enalapril pharmacokinetics in hypertensive pediatric patients (33).

Wells et al. (30) have divided the total number of hypertensive pediatric patients into four age groups Group I (1–24 months), Group II (25 months-6 years), Group III (6–12 years), Group IV (12–16 years) with 9–12 subjects in each group as summarized in Table 1. A single and multiple oral doses of 0.15 mg/kg of extemporaneously prepared suspension from crushed tablets were administered in the two youngest age groups in infants from 1 month up to children of 6 years of age. The two older age groups of patients above 6 years up to 16 years of age received single and multiple-dose of 2.5 mg of enalapril tablet daily for weight <28 kg and 5 mg daily for weight >28 kg. Nakamura et al. (31) have divided the heart failure pediatric patients based on age under 20 days and above 20 days. There were two neonates with three plasma concentration profiles below 20 days of age and 10 patients with 11 plasma concentration profiles receiving 0.05–0.30 mg/kg of extemporaneously prepared oral suspension from Renivace tablets (Banyu Pharmaceutical Co., Ltd, Tokyo, Japan). Nakamura et al. (31) also presented data from seven adult healthy volunteers who received 10 mg in form of enalapril tablet. Lloyd et al. (32) included all the 10 pediatric patients with heart failure in one age group of 6 weeks to 8 months. They administered an escalating dose of 0.02 mg/kg on day 1, 0.04 mg/kg on day 2, and 0.08 mg/kg on day 3 of enalapril oral aqueous solution prepared from lyophilized enalapril maleate powder (see also Tables 1, 2).

The sparsely sampled profiles in these studies have limited the detailed evaluation of the pharmacokinetics of enalapril and enalaprilat. A relatively richer serum sample profile was collected from hypertensive patients with 8 sampling points at 1, 2, 4, 6, 8, 12, 16, and 24 h on days 1 and 7. For patients younger than 4 years the sampling was sparsely collected at the predose and at 1, 4, 8, and 24 h. Sampling points adequately covered the elimination phase; however, the description of the absorption phases was hampered with number of sampling points. Nakamura et al. (31) collected only 5 serum samples starting at 2 h and then at 4, 8, 12, and 24 h post-dose from pediatric heart failure patients, with no rich sampling at the absorption and elimination phases. Lloyd et al. (32) collected samples at 4, 6, 8, and 24 h post-dose. Pharmacokinetic data were provided out of plasma concentrations. The half-life of accumulation for hypertensive patients was calculated from urinary elimination, while the half-life in pediatric heart failure patients was calculated from serum samples. The drug and metabolite profiles provided limited opportunity to calculate the *T*_max_ and half-life values, especially for heart failure patients. Bioanalysis was performed using a radioimmune assay (RIA) technique in all three studies. The RIA was not able to directly detect enalapril concentrations and thus were calculated as a difference between the total enalaprilat concentrations after hydrolysis of samples with crude enzyme of rat liver and the enalaprilat concentrations of the non-hydrolyzed sample (18, 34). In contrast, modern standard liquid chromatography-mass spectrometry methods directly detect enalapril and enalaprilat.

Enalapril pharmacokinetics results were sparsely reported in all three studies and are summarized in Table 1. Especially for the maximal concentration (*C*_max_) and time to maximal concentration (*T*_max_) no conclusive results could be delineated. The *C*_max_ values of enalapril were reported for hypertensive pediatric patients of all age groups and were not reported for heart failure patients. A *T*_max_ value of 1 h for enalapril was reported for hypertensive pediatric patients for all age groups. In the heart failure pediatric patients, the first sample taken was at 2-h post-dose; therefore, it failed to provide the drug levels at a 1-h post-dose to provide reliable *T*_max_ values. Llyod et al. (32) did not report *T*_max_ values for pediatric heart failure patients. For drug exposure, only Nakamura et al. (31) provided data in one large age group from 20 days up to 6.5 years of age. In addition, arithmetic means from 3 plasma profiles of two heart failure patients lower than 20 days of age were presented with 3-fold higher exposure than in the older age group. This few data indicated differences in exposure in neonates but were not sufficient to delineate any maturation or ontogeny process due to age.

Enalaprilat pharmacokinetic results were detailed relatively richer for hypertensive and heart failure patients and are summarized in Table 2. Wells et al. (30) reported that *C*_max_ values increased with age after single and multiple-dose of enalapril. Nakamura et al. (31) reported a 3-fold lower *C*_max_ value in pediatric heart failure patients compared to healthy adults. However, the less number of samples in the absorption phase of pediatric heart failure patients limited the reliability of the reported *C*_max_ values. *T*_max_ values in pediatric hypertensive patients had a substantial overlay and were more or less the same for all age groups. Time to maximal concentrations were reported to be later in the heart failure pediatric patients than in adult healthy volunteers indicating age and/or disease dependent differences in absorption or elimination. Nakamura et al. (31) and Wells et al. (30) have reported systemic exposure of enalaprilat, i.e., AUC per dose normalized for the bodyweight of 0.1 mg/kg and AUC per 0.15 mg/kg (ng*hr/mL) in pediatric heart failure patients, respectively. Llyod et al. (32) did not report AUC values.

For an illustration of the comparison of the pharmacokinetic data provided in the three studies, dose-normalized AUC values for the active metabolite enalaprilat were presented in Figure 2. For the Lloyd study, AUC values were calculated for comparison based on the mean profile after the 0.08 mg/kg dose. The comparison indicates that for children with hypertension enalaprilat exposure is lower in young children from 1 to 2 years compared to 12 to 16 years of age. In children with heart failure, the only two patients below 20 days of age showed a 5-fold higher exposure compared to the patients between 20 days and 6.5 years of age and healthy adults. The comparison of children with hypertension (1 month up to 6 years) (30) and heart failure (20 days to 6.5 years) (31), (6 weeks up to 8 months) (32) of similar age indicates that the dose-normalized exposure of enalaprilat is similar.

## Discussion

Our systematic literature search revealed that there is insufficient information to establish developmental pharmacokinetics of enalapril in pediatric patients with hypertension and heart failure. The number of patients included in the searched studies were limited and did not cover all the heterogeneous pediatric age groups. The pharmacokinetics of enalapril was under-reported in the literature and no information regarding the maturating route of the active metabolite enalaprilat could be delineated. The maturation in enalaprilat exposure was reported with age depicted by the AUC value. In pediatric patients with hypertension, maturation seemed to result in lower enalaprilat exposure in young compared to older patients. In pediatric patients with heart failure, however, the AUC values did not provide a clear picture of maturation in different age groups including neonates. A 5-fold higher value of enalaprilat exposure in only 2 pediatric patients at a very early age compared to patients above 20 days and adults warrant further clinical investigation in neonates with heart failure.

Enalapril pharmacokinetic results were not reported for pediatric hypertensive patients. In pediatric patients with heart failure, sparsely reported data demonstrated 3-fold higher enalapril exposure in 2 neonates with 3 plasma profiles of below 20 days of age. Whether this higher exposure can be due to the immature or disease-related eliminating organ function warrants further investigations. The reported enalapril half-life values or the information about the maximal time of enalapril plasma concentrations were limited due to the limited number of subjects and sampling points in the elimination and absorption phase of the investigated pediatric patients. In total, 5 plasma samples at 2, 4, 8, 12, and 24-h post-dose were collected which failed to sufficiently describe absorption and elimination phases of the drug.

The reported pharmacokinetic data sets of the active metabolite enalaprilat were richer than for enalapril. In pediatric patients with hypertension, a gradual increase in enalaprilat exposure was reported with increasing age and this points toward ontogeny of enalaprilat formation with maturating organ function. Because enalaprilat has a relatively high solubility in water, total body water presents a high part of the volume of distribution. Decrease of total body water (% of body weight) may explain a part of the trend of differences in exposure of enalaprilat over age. Enalaprilat exposure in the oldest pediatric age group was comparable to the enalaprilat exposure in hypertensive adults (25) and a similar exposure had been reported in adult patients with heart failure (35). This supports the fact that adult enalaprilat exposure data are higher in older than in younger patients and it indicates also that enalaprilat exposure in hypertensive and heart failure patients might not differ substantially. Whether neonates behave differently due to ontogeny or disease warrants further investigations since the number of three investigated plasma profiles of two patients below 20 days of age is too low to draw any conclusion.

The administered extemporaneously prepared suspensions (30, 31) or aqueous solution (32) in these pediatric studies may present dosing precision and compliance problems and may cause variability in pharmacokinetic parameters. However, due to sparsely reported enalapril data, the comparison of systemic exposure of enalapril against these formulations was not possible. The reported studies provided non-compartmental analyses with limited information related to the biometric, developmental and/or disease-related covariates on drug concentrations. Kechagia et al. (33), however, had performed a model-dependent analysis of the pediatric patient cohort with hypertension from Wells et al. (30) to predict the pharmacokinetic and pharmacodynamics (PKPD) of enalapril in the age group of 6–16 years and they further extrapolated the PKPD of enalapril for the age group 0–6 years. However, the study had several limitations as they only used mean digitized concentrations and accounted for the effect of body weight on clearance and volume of distribution without accounting the maturation with age using maturation function and used simulated datasets rather than a real clinical data set for the age group of 0–6 years.

## Conclusion

Data related to the pharmacokinetics of enalapril and enalaprilat in hypertensive patients and few data for young heart failure children are available. Comparison of dose normalized exposition of the active metabolite enalaprilat indicated similarities between heart failure and hypertensive patients and a potentially high exposition of premature patients but more pharmacokinetic data, especially in young patients with heart failure, are needed to assess the effect of age and disease and with this to provide a basis for a dosing regimen of enalapril in those pediatric patients (36). The current analysis has explained the trends in the pharmacokinetics of enalapril and enalaprilat in pediatric patients and in addition has highlighted the shortcomings in the reported clinical trials and sample collection that compromised proper pharmacokinetic analysis of the drug. This shall help in designing more informative clinical trials in the future for enalapril in pediatric patients. Different clinical trials are in process to establish safe and effective dose of enalapril in pediatric heart failure. One such study is the LENA (labeling of enalapril from neonates up to adolescents) clinical trials which aims at to analyze and establish the detailed developmental pharmacokinetics of enalapril and enalaprilat in pediatric patients with heart failure and dilated cardiomyopathy (37).

## Data Availability Statement

The raw data supporting the conclusions of this article will be made available by the authors, without undue reservation.

## Author Contributions

SL, WC, and MF contributed to the idea, drafting, and checking of this work. MF conducted the literature search and WC, SL did reevaluations. All authors contributed to the article and approved the submitted version.

## Conflict of Interest

The authors declare that the research was conducted in the absence of any commercial or financial relationships that could be construed as a potential conflict of interest.
